# Defective Iron Utilization in Heart Failure: A Prevalent Phenotype with Less Frequent Iron Therapy

**DOI:** 10.3390/nu18213515

**Published:** 2026-10-26

**Authors:** Mats Kutscher, Wendimagegn Alemayehu, Nicolò de Biase, Julia Benjamins, Ryan Vos, Marieke C. Haan, Cynthia M. Westerhout, Justin Ezekowitz, Niels Grote Beverborg

**Affiliations:** 1 Department of Cardiology, University Medical Centre Groningen, 9713 GZ Groningen, The Netherlands; m.kutscher@umcg.nl (M.K.);; 2 Canadian VIGOUR Centre, University of Alberta, Edmonton, AB T6G 2E1, Canada; 3 Department of Clinical and Experimental Medicine, University of Pisa, 56126 Pisa, Italy; 4 Division of Cardiology, University of Alberta, Edmonton, AB T6G 2B7, Canada

**Keywords:** heart failure, iron deficiency, defective iron utilization, low iron storage, iron therapy, intravenous iron

## Abstract

Background/Objectives: Iron deficiency (ID) in heart failure (HF) can be classified as low iron storage (LIS) or defective iron utilization (DIU). Whether these phenotypes differ in clinical characteristics, outcomes, and treatment patterns in a real-world decompensated HF population remains unclear. Methods: In this population-based retrospective cohort study, 4818 adults with decompensated HF and available iron indices were classified as LIS (TSAT < 20%, ferritin < 100 µg/L), DIU (TSAT < 20%, ferritin ≥ 100 µg/L), or no ID (TSAT ≥ 20%). All-cause mortality, cardiovascular (CV) hospitalization, HF hospitalization, and initiation of iron therapy were assessed over a median follow-up of 24 months. Outcomes were analyzed using Cox proportional hazards models adjusted for pre-specified covariates, with Fine–Gray competing-risk models for hospitalization and iron therapy initiation, and no ID as the reference group. Results: DIU was present in 30.2% of patients, compared with 38.7% with LIS and 31.1% with no ID. Iron therapy was initiated in 21.5% of patients with DIU compared with 32.0% with LIS, and intravenous (IV) iron in 2.7% versus 4.3%, respectively. After adjustment, DIU was associated with modestly higher CV hospitalization rates (aHR 1.15; 95% CI 1.03–1.28) and with the composite of CV hospitalization or all-cause mortality (aHR 1.09; 95% CI 1.00–1.19), but not with all-cause mortality (aHR 1.03; 95% CI 0.93–1.14) or HF hospitalization (aHR 1.10; 95% CI 0.93–1.27) compared with no ID. LIS was associated with higher risk of HF hospitalization (aHR 1.38; 95% CI 1.19–1.59). Patients with LIS were more likely to initiate iron therapy than those with DIU (adjusted sHR 1.49; 95% CI 1.29–1.71). Conclusions: DIU is a prevalent phenotype of ID in decompensated HF that is less frequently treated with iron than LIS, despite a largely similar prognosis, and is associated with modestly higher CV hospitalization and the composite of CV hospitalization or death, but not independently with mortality. Prospective evaluation of iron replacement strategies, particularly IV iron, in patients with DIU is warranted.

## 1. Introduction

Iron deficiency (ID) affects approximately 50% of patients with heart failure (HF) and is an independent predictor of adverse outcomes [1]. Iron is essential for oxygen transport and mitochondrial function in all cells including cardiomyocytes, where ID directly impairs contractility through reduced mitochondrial respiration and ATP production [2]. ID can be caused by decreased intake or absorption, abnormal metabolism or increased losses. To stratify between potential etiologies in patients with ID (defined as transferrin saturation [TSAT] <20%), the distinction is often made between low iron storage (LIS; ferritin < 100 µg/L) and defective iron utilization (DIU; ferritin ≥ 100 µg/L). DIU may arise from hepcidin-driven sequestration of iron in macrophages and enterocytes, rendering otherwise adequate stores unavailable for erythropoiesis and cellular metabolism [3]. A prior study examined clinical differences between LIS and DIU, reporting that LIS, but not DIU, was independently associated with higher mortality and hospitalization rates in selected HF cohorts [3]. Whether these findings generalize to a real-world population, and how treatment patterns differ between phenotypes, remains unknown. Distinguishing between these phenotypes is clinically relevant, as the underlying mechanisms may predict differential responses to iron repletion. We therefore investigated the prevalence, clinical profiles, prognosis, and iron therapy initiation of DIU compared to LIS and no ID in a large population-based decompensated HF cohort.

## 2. Materials and Methods

Data were obtained from linked administrative health databases maintained by Alberta Health Services and Alberta Health as previously described [4]. The cohort comprised adult patients with an emergency department visit or hospitalization with a primary HF diagnosis (ICD-10 I50.x) between 1 May 2012 and 31 March 2019; the index date was the first visit. Patients were followed until 31 March 2020, death, or outmigration. Iron indices (TSAT and ferritin) measured within 30 days of the index episode were used to classify patients as LIS, DIU, or no ID. Patients with missing iron indices that are needed for classification were not included (Figure S1). ID was defined as TSAT < 20%, based on the threshold validated against bone marrow iron staining [5]; LIS and DIU were subsequently distinguished by a ferritin threshold of 100 µg/L, the most widely used clinical cutoff, which approximates the bone marrow-validated cutoff of 128 µg/L [3]. A sensitivity analysis using 128 µg/L was not performed, because this cutoff derives from a small bone marrow study (n = 42). Comorbidities were identified from ICD-10 codes over the five years preceding the index episode. HF subtype was based on left ventricular ejection fraction (LVEF), which was available in all patients: HFrEF (LVEF < 40%), HFmrEF (40–49%), and HFpEF (≥50%). Hospitalization events over the follow-up period were captured based on ICD-10 codes. CV hospitalization was defined as any hospitalization with a primary CV diagnosis (ICD-10 I00–I99), which includes HF hospitalization; the two outcomes are therefore not mutually exclusive. The composite endpoint was the time to the first CV hospitalization or all-cause death. Iron supplementation therapy (oral or intravenous [IV]) was assessed over the entire follow-up period through hospital and pharmacy dispensations; patients were not excluded based on prior iron use.

Time to the first event of all-cause mortality, CV hospitalization, HF hospitalization, and the composite of CV hospitalization or all-cause mortality was analyzed using Cox proportional hazards models. CV and HF hospitalization were additionally analyzed using Fine–Gray competing-risk models with death as the competing event. Initiation of iron therapy was analyzed using a Fine–Gray competing-risk model with death without prior iron therapy as the competing event. A total of 22 patients were lost to follow-up due to outmigration and were censored at the time of migration if no event was observed. All models were adjusted for a pre-specified set of covariates based on the independent determinants of ID in HF reported by Van der Wal et al. [6]: age, sex, hemoglobin, antiplatelet use, and renal disease (binary). Age and hemoglobin were flexibly modeled using splines. The proportional hazards assumption was assessed for all covariates using time-dependent covariate terms; age violated the assumption and was therefore modeled as a time-varying covariate. Adjusted analyses were complete-case (effective sample size n = 4816). Adjusted hazard ratios (aHRs), adjusted subdistribution hazard ratios (sHRs), and 95% confidence intervals (CIs) are reported. Effect modification by age, sex, coronary artery disease, and HF type was explored using interaction terms. Patients with and without iron testing were compared (Table S1). No correction for multiple comparisons was applied across outcomes, which are clinically distinct, and associations with borderline *p*-values should be interpreted with caution. All analyses were performed in SAS (version 9.4). Ethics approval was obtained from the University of Alberta (Pro00010852).

## 3. Results

Of 17,463 patients with decompensated HF identified between May 2012 and March 2019, 6730 (38.5%) had iron indices measured within 30 days of the index episode, and 4818 (27.6%) had indices that allowed classification and were included (Figure S1): 1498 (31.1%) had no ID, 1865 (38.7%) had LIS, and 1455 (30.2%) had DIU. Patients with iron testing were younger (median 75 vs. 77 years) and more often treated in tertiary hospitals (33.1% vs. 23.9%) than those without, and had more diabetes (49.6% vs. 41.6%), anemia (41.9% vs. 20.3%), and renal disease (35.5% vs. 21.5%; Table S1). Iron indices were measured a median of 4 days before discharge from the index episode (IQR 15 days before to 4 days after); 66.0% of measurements were taken before and 34.0% after discharge.

Baseline patient characteristics are presented in Table 1. Patients with DIU were younger (median 72 years) than those with LIS (77 years) and no ID (75 years) and were more often male than those with LIS(58.5% vs. 46.3% and 59.9%, respectively). Patients with LIS had the highest comorbidity burden, including anemia (52.0% vs. 44.2% in DIU and 38.9% in no ID), atrial fibrillation (52.5% vs. 41.5% and 47.1%), and COPD (35.1% vs. 29.2% and 31.0%). Median NT-proBNP was highest in DIU (3975 pg/mL), lowest in LIS (2723 pg/mL), and intermediate in no ID (3377 pg/mL), whereas renal disease was most prevalent in no ID (46.0%), followed by DIU (41.9%) and LIS (32.9%). The proportion of HFrEF was similar across groups (30.8% in no ID, 26.6% in LIS, and 29.3% in DIU).

Over a median follow-up of 24 months, DIU was associated with higher CV hospitalization (aHR 1.15; 95% CI 1.03–1.28) compared with no ID. The association with the composite of CV hospitalization or all-cause mortality was significant before adjustment (HR 1.11; 95% CI 1.02–1.21) and after adjustment (aHR 1.09; 95% CI 1.00–1.19; *p* = 0.11 across groups), whereas associations with all-cause mortality (aHR 1.03; 95% CI 0.93–1.14) and HF hospitalization (aHR 1.10; 95% CI 0.93–1.27) were not significant (Table 2). LIS was associated with higher HF hospitalization (aHR 1.38; 95% CI 1.19–1.59) and, to a lesser extent than DIU, CV hospitalization (aHR 1.11; 95% CI 1.00–1.24), but not with all-cause mortality (aHR 0.95; 95% CI 0.86–1.05). Fine–Gray analyses with death as the competing event gave consistent results for CV hospitalization (LIS sHR 1.12, 95% CI 1.01–1.25; DIU sHR 1.13, 95% CI 1.02–1.27) and HF hospitalization (LIS sHR 1.39, 95% CI 1.20–1.61; DIU sHR 1.09, 95% CI 0.93–1.27). No significant interactions were observed between ID type and age, sex, coronary artery disease, or HF type for the composite outcome in unadjusted and adjusted models (all *p*-interaction >0.05).

**Figure 1:**
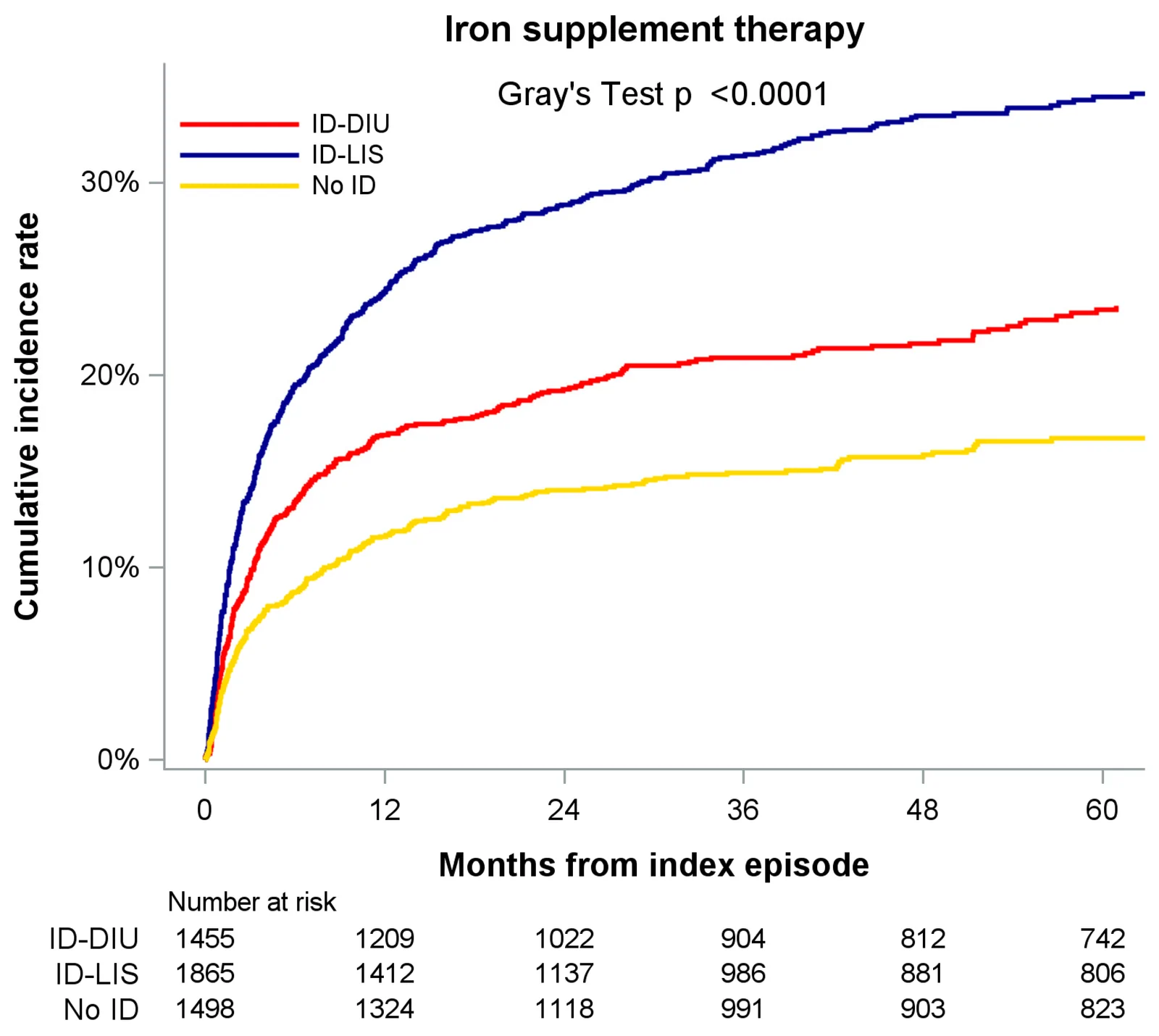
Cumulative incidence of iron therapy initiation following the index episode according to iron deficiency type. Abbreviations: DIU, defective iron utilization; HF, heart failure; ID, iron deficiency; LIS, low iron storage. Cumulative incidence curves accounting for the competing risk of death without prior iron therapy. ID-DIU (red), ID-LIS (blue), no ID (yellow). Gray’s test *p* < 0.0001. Curves represent unadjusted cumulative incidence; adjusted estimates are presented in Table 2. Number at risk refers to patients who had not yet initiated iron therapy and had not died at each time point.

Over the follow-up period, iron therapy was initiated in 32.0% of patients with LIS and 21.5% of patients with DIU. IV iron was used in only 4.3% and 2.7% respectively, with oral bivalent iron predominating in both groups. Figure 1 shows the cumulative incidence of iron therapy initiation over time, demonstrating that patients with LIS started therapy earlier and more frequently than both DIU and no ID patients (Gray’s test *p* < 0.0001). In a competing-risk analysis adjusted for pre-specified covariates, patients with LIS were more likely to initiate iron therapy earlier than both no ID (adjusted sHR 1.92; 95% CI 1.65–2.25) and DIU (adjusted sHR 1.49; 95% CI 1.29–1.71), and patients with DIU were more likely than those with no ID to initiate iron therapy (adjusted sHR 1.29; 95% CI 1.09–1.53; Table 2).

## 4. Discussion

This population-based study shows that DIU is a prevalent phenotype of ID in decompensated HF that is less frequently treated with iron than LIS. Patients with DIU were younger, more often male, and had higher NT-proBNP. After adjustment for pre-specified covariates, DIU was associated with modestly higher CV hospitalization, whereas no independent association with all-cause mortality or HF hospitalization was observed. Patients with LIS, characterized by older age, female predominance, more prevalent HFpEF, and high comorbidity burden, had a higher risk of HF hospitalization and, to a lesser extent, CV hospitalization. Neither phenotype was independently associated with all-cause mortality.

These findings partly agree with the only prior study in this area [3], which reported that LIS, but not DIU, was independently associated with higher mortality and HF hospitalization rates: we confirm the association of LIS with HF hospitalization, but did not observe an association with mortality. Although our formal interaction analysis did not show significant effect modification by HF type (all *p*-interaction > 0.05), possibly due to limited statistical power for subgroup analyses, the present cohort comprises predominantly HFpEF patients presenting with decompensated HF, whereas the prior study enrolled predominantly HFrEF patients with worsening HF. This compositional difference may partly contribute to the divergent findings, as may differences in covariate adjustment and in data capture between trial and administrative databases. The high hospitalization rate in patients with LIS, despite lower NT-proBNP and less renal disease, potentially reflects comorbidity-driven admissions independent of cardiac status. 

Patients with DIU were substantially less likely than those with LIS to receive iron therapy. Whether this reflects diagnostic oversight, with clinicians interpreting elevated ferritin as adequate iron stores, or a more conservative approach in patients perceived as too ill to benefit remains unclear. Oral bivalent iron, which predominated in this cohort, is mechanistically suboptimal in patients with DIU given hepcidin-mediated intestinal absorption blockade [7]. IV iron is guideline-recommended for patients with HFrEF and ID [8] supported by a recent meta-analysis demonstrating a 28% reduction in recurrent HF hospitalizations and cardiovascular mortality at 12 months [9].

The majority of patients in the present cohort had HFpEF, for which direct trial evidence is lacking. However, HFpEF was somewhat more prevalent in LIS (60.6%) than in DIU (55.5%), so the HF phenotype does not explain the lower treatment rate in DIU. Whether oral iron provides benefit in patients with DIU and whether IV iron specifically improves prognosis in this phenotype warrant prospective investigation. Some patients classified as no ID may have received iron for non-cardiac indications, or may have had TSAT normalized by supplementation already in use at the time of measurement.

Limitations include the absence of inflammatory markers or hepcidin, precluding exclusion of inflammatory ferritin elevation as a driver of DIU classification. Elevated hepcidin levels have previously been reported in patients with DIU [3], supporting this mechanistic pathway, although this was demonstrated in a predominantly HFrEF population. The ferritin cutoff of 100 µg/L provides a practical separation between LIS and DIU, but its precise sensitivity and specificity against bone marrow iron staining remain unclear [5].

Approximately 17% of patients had oral iron dispensed before the index episode, which may have influenced iron indices at classification. Iron indices were available in a minority of the source population, and tested patients had a higher comorbidity burden (Table S1). Together with the single-province setting, with its own healthcare system and iron prescribing patterns, this limits generalizability. Analyses were complete-case, and residual confounding, for example by HF severity, cannot be excluded. The administrative database design limits availability of clinical parameters such as NYHA class. Furthermore, hemoglobin levels were low across all groups, reflecting the overall high disease burden of this administratively identified cohort and highlighting the inherent selection toward more complex patients in whom iron indices were measured.

In conclusion, DIU is a prevalent phenotype of ID in decompensated HF that is less frequently treated with iron than the LIS phenotype, despite a largely similar prognosis and a somewhat lower HFpEF prevalence. DIU is associated with modestly higher CV hospitalization and the composite of CV hospitalization or death compared with no ID, but not independently with mortality. Increased clinical awareness of DIU and dedicated prospective trials of IV iron therapy in this population are warranted.

## Figures and Tables

**Table 1:** Baseline characteristics of patients with heart failure categorized by iron deficiency status.

Baseline Characteristics	No Iron Deficiency	Low Iron Storage (LIS)	Defective Iron Utilization (DIU)	*p*-Value
Total N	1498	1865	1455	
**Age (median, IQR)**	75 (64, 83)	77 (67, 84)	72 (62, 82)	<0.0001
**Sex**				<0.0001
Female	600 (40.1)	1002 (53.7)	604 (41.5)	
Male	898 (59.9)	863 (46.3)	851 (58.5)	
**Hospital type**				0.53
Non-tertiary	984 (65.7)	1216 (65.2)	928 (63.8)	
Tertiary hospital	514 (34.3)	649 (34.8)	527 (36.2)	
**HF type**				0.0078
HFmrEF	206 (13.8)	239 (12.8)	220 (15.1)	
HFpEF	830 (55.4)	1130 (60.6)	808 (55.5)	
HFrEF	462 (30.8)	496 (26.6)	427 (29.3)	
**Comorbidities**				
Diabetes	744 (49.7)	999 (53.6)	734 (50.4)	0.054
Hypertension	1306 (87.2)	1633 (87.6)	1250 (85.9)	0.36
Hyperlipidemia	250 (16.7)	341 (18.3)	258 (17.7)	0.48
CAD	824 (55.0)	1040 (55.8)	744 (51.1)	0.021
PVD	277 (18.5)	345 (18.5)	249 (17.1)	0.52
Atrial fibrillation	706 (47.1)	979 (52.5)	604 (41.5)	<0.0001
Stroke/TIA	203 (13.6)	296 (15.9)	197 (13.5)	0.082
Thromboembolism	232 (15.5)	306 (16.4)	256 (17.6)	0.30
Asthma	132 (8.8)	197 (10.6)	136 (9.3)	0.21
COPD	464 (31.0)	655 (35.1)	425 (29.2)	0.0008
Anemia	583 (38.9)	970 (52.0)	643 (44.2)	<0.0001
Cancer	255 (17.0)	309 (16.6)	243 (16.7)	0.94
Liver disease	125 (8.3)	128 (6.9)	110 (7.6)	0.27
Renal disease	689 (46.0)	614 (32.9)	609 (41.9)	<0.0001
Depression	285 (19.0)	425 (22.8)	273 (18.8)	0.0048
Dementia	118 (7.9)	202 (10.8)	120 (8.2)	0.0047
Charlson Comorbidity Index	5 (3, 7)	5 (3, 7)	5 (3, 7)	0.83
LVEF	53 (35, 65)	55 (39, 65)	53 (36, 65)	0.0071
**Baseline medications**				
ACEi/ARB/ARNI	1035 (69.1)	1337 (71.7)	1033 (71.0)	0.25
Beta blocker	995 (66.4)	1256 (67.3)	930 (63.9)	0.11
MRA	229 (15.3)	297 (15.9)	192 (13.2)	0.080
Digoxin	87 (5.8)	150 (8.0)	92 (6.3)	0.025
Statin	861 (57.5)	1128 (60.5)	874 (60.1)	0.18
Anticoagulant	532 (35.5)	846 (45.4)	464 (31.9)	<0.0001
Antiplatelet	518 (34.6)	735 (39.4)	563 (38.7)	0.0103
**Baseline iron-related therapy**				
Erythropoiesis stimulating	28 (1.9)	6 (0.3)	10 (0.7)	<0.0001
Darbepoetin alpha	204 (13.6)	37 (2.0)	133 (9.1)	<0.0001
Oral iron	249 (16.6)	310 (16.6)	261 (17.9)	0.54
Parenteral iron	4 (0.3)	10 (0.5)	5 (0.3)	0.43
Other iron supplement	23 (1.5)	28 (1.5)	12 (0.8)	0.15
**Baseline lab results**				
Serum iron (µmol/L)	13 (11, 17)	5 (4, 7)	6 (4, 8)	<0.0001
Ferritin (µg/L)	212 (107, 452); 85%	42 (24, 64)	201 (136, 348)	<0.0001
TSAT (%)	26.7 (22.9, 35.2)	8.8 (5.9, 12.5)	12.8 (9.4, 16.2)	<0.0001
Hemoglobin (g/dL)	11.4 (9.8, 13.4); 99.9%	10.3 (9.2, 11.8)	10.7 (9.4, 12.4)	<0.0001
Serum creatinine (µmol/L)	126 (90, 235); 98.2%	109 (82, 154); 99.2%	124.5 (87, 217); 99.4%	<0.0001
eGFR (mL/min/1.73 m^2^)	46 (21, 66); 82.5%	54 (35, 71); 77.6%	48 (24, 69); 79.6%	<0.0001
BNP (pg/mL)	944 (557, 1817); 21.9%	865 (472.5, 1630); 29.2%	911 (455, 1711); 27.4%	0.26
NT-proBNP (pg/mL)	3377 (1401, 8908); 52.8%	2723 (1077, 6649); 51.6%	3975 (1620, 10,374); 52.2%	<0.0001
**Iron supplementation post-index**				
No therapy, n (%)	1261 (84.2)	1268 (68.0)	1142 (78.5)	
Any type *, n (%)	237 (15.8)	597 (32.0)	313 (21.5)	
Oral therapy	214 (14.3)	538 (28.8)	285 (19.6)	
IV therapy	30 (2.0)	81 (4.3)	39 (2.7)	

Abbreviations: ACEi, angiotensin-converting enzyme inhibitor; ARB, angiotensin receptor blocker; ARNI, angiotensin receptor-neprilysin inhibitor; CAD, coronary artery disease; COPD, chronic obstructive pulmonary disease; DIU, defective iron utilization; eGFR, estimated glomerular filtration rate; HF, heart failure; HFmrEF, heart failure with mildly reduced ejection fraction; HFpEF, heart failure with preserved ejection fraction; HFrEF, heart failure with reduced ejection fraction; ID, iron deficiency; IQR, interquartile range; IV, intravenous; LIS, low iron storage; LVEF, left ventricular ejection fraction; MRA, mineralocorticoid receptor antagonist; NT-proBNP, N-terminal pro-B-type natriuretic peptide; PVD, peripheral vascular disease; TSAT, transferrin saturation. * There are some patients receiving both oral and IV therapy. Values presented as median (IQR); percentage indicates data completeness where <100%. *p*-values reflect the overall difference across the three groups (chi-squared test for categorical variables; Kruskal–Wallis test for continuous variables). Pairwise post hoc tests were not performed.

**Table 2:** Clinical outcomes and iron therapy initiation over a median 24-month follow-up in patients with heart failure, stratified by iron deficiency status.

Outcome	Without ID	ID-LIS	ID-DIU	*p*-Value
**Total N**	1498	1865	1455	
CV hosp./mortality *				
Pts with events, n (%)	1055 (70.4%)	1327 (71.2%)	1061 (72.9%)	
# events/100 patient-years	38.6	42.3	44.2	
Unadjusted HR (95% CI)	Reference	1.06 (0.98–1.15)	1.11 (1.02–1.21)	0.043
Adjusted * HR (95% CI)	Reference	1.03 (0.94–1.12)	1.09 (1.00–1.19)	0.11
All-cause mortality *				
Pts with events, n (%)	780 (52.1%)	983 (52.7%)	771 (53.0%)	
# events/100 patient-years	20.1	21.5	20.8	
Unadjusted HR (95% CI)	Reference	1.05 (0.96–1.16)	1.03 (0.93–1.14)	0.54
Adjusted * HR (95% CI)	Reference	0.95 (0.86–1.05)	1.03 (0.93–1.14)	0.28
CV hosp. *				
Pts with events, n (%)	625 (41.7%)	828 (44.4%)	666 (45.8%)	
# events/100 patient-years	22.9	26.4	27.8	
Unadjusted HR (95% CI)	Reference	1.11 (1.00–1.23)	1.17 (1.05–1.31)	0.015
Adjusted * HR (95% CI)	Reference	1.11 (1.00–1.24)	1.15 (1.03–1.28)	0.043
Adjusted ° sHR (95% CI)	Reference	1.12 (1.01–1.25)	1.13 (1.02–1.27)	0.045
HF hosp. *				
Pts with events, n (%)	320 (21.4%)	536 (28.7%)	335 (23.0%)	
# events/100 patient-years	9.3	14.3	10.4	
Unadjusted HR (95% CI)	Reference	1.44 (1.26–1.66)	1.10 (0.94–1.28)	<0.001
Adjusted * HR (95% CI)	Reference	1.38 (1.19–1.59)	1.10 (0.93–1.27)	<0.001
Adjusted ° sHR (95% CI)	Reference	1.39 (1.20–1.61)	1.09 (0.93–1.27)	<0.001
**Time to iron therapy initiation °**				
Patients initiating, n (%)	237 (15.8)	597 (32.0)	313 (21.5)	
# events/100 patient-years	7.1	17.9	10.5	
Unadjusted sHR (95% CI)	Reference	2.25 (1.94–2.62)	1.41 (1.19–1.67)	<0.001
Adjusted sHR (95% CI)	Reference	1.92 (1.65–2.25)	1.29 (1.09–1.53)	<0.001
**Iron therapy initiation: LIS vs. DIU °**				
Unadjusted sHR (95% CI)		1.60 (1.39–1.82)	Reference	<0.001
Adjusted sHR (95% CI)		1.49 (1.29–1.71)	Reference	<0.001

Abbreviations: CI, confidence interval; CV, cardiovascular; DIU, defective iron utilization; HF, heart failure; (s)HR, (subdistribution) hazard ratio; ID, iron deficiency; LIS, low iron storage. * Cox proportional hazards model adjusted for age (time-varying), sex, hemoglobin, antiplatelet use, and renal disease; n = 4816 with complete covariate data. *p*-values reflect the overall difference across the three groups from the Cox or Fine–Gray model. Median (IQR) follow-up: 24 (12.3–45.3) months. ° Fine–Gray competing-risk model with the same covariates; death was the competing event for hospitalization outcomes, and death without prior iron therapy for iron therapy initiation. # = number of.

## Data Availability

The data used in this study are not publicly available because they are derived from administrative health databases maintained by Alberta Health Services and Alberta Health and are subject to privacy and data access restrictions.

## Supplementary Materials

The following supporting information can be downloaded at https://www.mdpi.com/article/10.3390/nu18213515/s1: Figure S1: Patient flow chart; Table S1: Baseline characteristics of patients with and without iron testing.

## Abbreviations

The following abbreviations are used in this manuscript: ACEi, angiotensin-converting enzyme inhibitor; ARB, angiotensin receptor blocker; ARNI, angiotensin receptor-neprilysin inhibitor; CAD, coronary artery disease; COPD, chronic obstructive pulmonary disease; DIU, defective iron utilization; eGFR, estimated glomerular filtration rate; HF, heart failure; HFmrEF, heart failure with mildly reduced ejection fraction; HFpEF, heart failure with preserved ejection fraction; HFrEF, heart failure with reduced ejection fraction; ID, iron deficiency; IQR, interquartile range; IV, intravenous; LIS, low iron storage; LVEF, left ventricular ejection fraction; MRA, mineralocorticoid receptor antagonist; NT-proBNP, N-terminal pro-B-type natriuretic peptide; PVD, peripheral vascular disease; TSAT, transferrin saturation.

## References

[1] Klip I.T., Comin-Colet J., Voors A.A., Ponikowski P., Enjuanes C., Banasiak W., Lok D.J., Rosentryt P., Torrens A., Polonski L. (2013). Iron deficiency in chronic heart failure: An international pooled analysis. Am. Heart J..

[2] Hoes M.F., Grote Beverborg N., Kijlstra J.D., Kuipers J., Swinkels D.W., Giepmans B.N.G., Rodenburg R.J., Van Veldhuisen D.J., De Boer R.A., Van Der Meer P. (2018). Iron Deficiency Impairs Contractility of Human Cardiomyocytes Through Decreased Mitochondrial Function. Eur. J. Heart Fail..

[3] Beverborg N.G., van der Wal H.H., Klip I.T., Anker S.D., Cleland J., Dickstein K., van Veldhuisen D.J., Voors A.A., van der Meer P. (2019). Differences in Clinical Profile and Outcomes of Low Iron Storage vs. Defective Iron Utilization in Patients with Heart Failure. JAMA Cardiol..

[4] Wahid M., Islam S., Sepehrvand N., Dover D.C., McAlister F.A., Kaul P., Ezekowitz J.A. (2024). Iron Deficiency, Anemia, and Iron Supplementation in Patients with Heart Failure: A Population-Level Study. Circ. Heart Fail..

[5] Beverborg N.G., Klip I.T., Meijers W.C., Voors A.A., Vegter E.L., van der Wal H.H., Swinkels D.W., van Pelt J., Mulder A.B., Bulstra S.K. (2018). Definition of Iron Deficiency Based on the Gold Standard of Bone Marrow Iron Staining in Heart Failure Patients. Circ. Heart Fail..

[6] van der Wal H.H., Beverborg N.G., Dickstein K., Anker S.D., Lang C.C., Ng L.L., van Veldhuisen D.J., A Voors A., van der Meer P. (2019). Iron deficiency in worsening heart failure is associated with reduced estimated protein intake, fluid retention, inflammation, and antiplatelet use. Eur. Heart J..

[7] Martens P., Beverborg N.G., van der Meer P. (2021). Iron deficiency in heart failure—Time to redefine. Eur. J. Prev. Cardiol..

[8] Køber L., Adamo M., Ruwald A.-C., Tomasoni D., Anderson L.J., Andersson C., Brugts J.J., Chioncel O., Donal E., Ferdinandy P. (2026). 2026 ESC Guidelines for the management of heart failure. Eur. Heart J..

[9] Anker S.D., Karakas M., Mentz R.J., Ponikowski P., Butler J., Khan M.S., Talha K.M., Kalra P.R., Hernandez A.F., Mulder H. (2025). Systematic review and meta-analysis of intravenous iron therapy for patients with heart failure and iron deficiency. Nat. Med..

