# Supplementary material for: Defective Iron Utilization in Heart Failure: A Prevalent Phenotype with Less Frequent Iron Therapy
Source: Nutrients. 2026 Oct 26;18(21):3515. doi: 10.3390/nu18213515 (PMC13765118; doi:10.3390/nu18213515)
Supplement: Supplementary file 1 [file nutrients-18-03515-s001.zip › nutrients-4582665-supplementary.pdf]

Supplementary Materials

Defective iron utilization in heart failure: a prevalent phenotype with less frequent iron therapy

Contents

Figure S1. Patient flow chart
Table S1. Baseline characteristics of patients with and without iron testing

Figure S1. Patient flow chart.

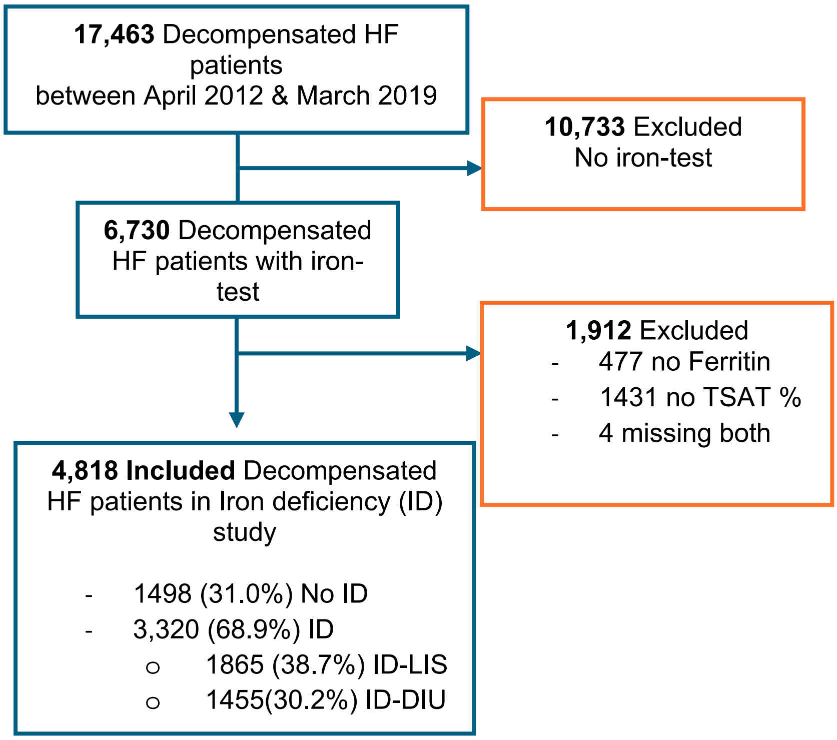

Abbreviations: DIU, defective iron utilization; HF, heart failure; ID, iron deficiency; LIS, low iron storage; TSAT, transferrin saturation. ED, emergency department.

Table S1. Baseline characteristics of patients with and without iron testing.

Characteristics	*Statistic*	*Not tested*	*Tested*	*Total*	*p-value*
Total N	*N*	*10,733*	*6,730*	*17,463*	
Age	*Median (IQR)*	*77 (67, 85)*	*75 (65, 84)*	*77 (66, 85)*	*<.0001*
Sex-male	*n (%)*	*5,848 (54.5)*	*3,643 (54.1)*	*9,491 (54.3)*	*0.6463*
Residence-Rural	*n (%)*	*1,852 (17.3)*	*1,028 (15.3)*	*2,880 (16.5)*	*0.0006*
Tertiary hospital	*n (%)*	*2,563 (23.9)*	*2,227 (33.1)*	*4,790 (27.4)*	
HF type					
- ***mEF***	*n (%)*	*1,509 (14.1)*	*930 (13.8)*	*2,439 (14.0)*	*0.8693*
- ***pEF***	*n (%)*	*6,134 (57.2)*	*3,844 (57.1)*	*9,978 (57.1)*	
- ***rEF***	*n (%)*	*3,090 (28.8)*	*1,956 (29.1)*	*5,046 (28.9)*	
Diabetes	*n (%)*	*4,464 (41.6)*	*3,335 (49.6)*	*7,799 (44.7)*	*<.0001*
Hypertension	*n (%)*	*9,183 (85.6)*	*5,826 (86.6)*	*15,009 (85.9)*	*0.0619*
Hyperlipidemia	*n (%)*	*1,765 (16.4)*	*1,138 (16.9)*	*2,903 (16.6)*	*0.4221*
CAD	*n (%)*	*5,721 (53.3)*	*3,604 (53.6)*	*9,325 (53.4)*	*0.7488*
PVD	*n (%)*	*1,604 (14.9)*	*1,164 (17.3)*	*2,768 (15.9)*	*<.0001*
Atrial Fibrillation	*n (%)*	*5,822 (54.2)*	*3,253 (48.3)*	*9,075 (52.0)*	*<.0001*
Stroke/TIA	*n (%)*	*1,612 (15.0)*	*969 (14.4)*	*2,581 (14.8)*	*0.2605*
Thromboembolism	*n (%)*	*1,623 (15.1)*	*1,105 (16.4)*	*2,728 (15.6)*	*0.0215*
Asthma	*n (%)*	*1,089 (10.1)*	*654 (9.7)*	*1,743 (10.0)*	*0.3578*
CPOD	*n (%)*	*3,809 (35.5)*	*2,174 (32.3)*	*5,983 (34.3)*	*<.0001*
Anemia	*n (%)*	*2,174 (20.3)*	*2,820 (41.9)*	*4,994 (28.6)*	*<.0001*
Cancer	*n (%)*	*1,741 (16.2)*	*1,168 (17.4)*	*2,909 (16.7)*	*0.0503*
Liver disease	*n (%)*	*419 (3.9)*	*474 (7.0)*	*893 (5.1)*	*<.0001*
Renal disease	*n (%)*	*2,306 (21.5)*	*2,386 (35.5)*	*4,692 (26.9)*	*<.0001*
Depression	*n (%)*	*2,091 (19.5)*	*1,361 (20.2)*	*3,452 (19.8)*	*0.2315*
Dementia	*n (%)*	*1,116 (10.4)*	*634 (9.4)*	*1,750 (10.0)*	*0.0363*
Charlson Comorbidity Index	*Median (IQR)*	*4 (2, 6)*	*5 (3, 7)*	*4 (2, 6)*	*<.0001*
LVEF	*Median (IQR)*	*54 (37, 65)*	*53 (37, 65)*	*54 (37, 65)*	*0.9766*
ACEi/ARB/ARNI	*n (%)*	*7,713 (71.9)*	*4,757 (70.7)*	*12,470 (71.4)*	*0.0933*
Beta blocker	*n (%)*	*7,264 (67.7)*	*4,433 (65.9)*	*11,697 (67.0)*	*0.0133*
MRA	*n (%)*	*1,812 (16.9)*	*1,039 (15.4)*	*2,851 (16.3)*	*0.012*
Digoxin	*n (%)*	*967 (9.0)*	*483 (7.2)*	*1,450 (8.3)*	*<.0001*
Statin	*n (%)*	*5,902 (55.0)*	*3,929 (58.4)*	*9,831 (56.3)*	*<.0001*
Erythropoeitin	*n (%)*	*19 (0.2)*	*54 (0.8)*	*73 (0.4)*	*<.0001*

Darbepoetin alpha	*n (%)*	*97 (0.9)*	*417 (6.2)*	*514 (2.9)*	*<.0001*
Oral Iron	*n (%)*	*1,024 (9.5)*	*1,091 (16.2)*	*2,115 (12.1)*	*<.0001*
Parenteral Iron	*n (%)*	*22 (0.2)*	*27 (0.4)*	*49 (0.3)*	*0.017*
Other Iron supplement	*n (%)*	*77 (0.7)*	*82 (1.2)*	*159 (0.9)*	*0.0007*

Values are n (%) unless stated otherwise. "Tested" refers to patients with at least one iron test within 30 days of the index episode (n=6,730), of whom 4,818 had indices that allowed classification and were included in the analysis. p-values compare tested with not tested patients.
Abbreviations: ACEi, angiotensin-converting enzyme inhibitor; ARB, angiotensin receptor blocker; ARNI, angiotensin receptor-neprilysin inhibitor; COPD, chronic obstructive pulmonary disease; HF, heart failure; HFmrEF, HF with mildly reduced ejection fraction; HFpEF, HF with preserved ejection fraction; HFrEF, HF with reduced ejection fraction; IQR, interquartile range; LVEF, left ventricular ejection fraction; MRA, mineralocorticoid receptor antagonist; TIA, transient ischemic attack.
